# Physical Culture and Spinal Curvature

**Published:** 1906-07-07

**Authors:** H. R. Heather Bigg


### Hospital Clinics.

### PHYSICAL CULTURE AND SPINAL CURVATURE.

By H. R. Heather Bigg, F.R.C.S.Edin.

The fact that the healthy human body can be brought to its fullest strength by regulated physical exercises has been well known in all ages. The ancient Greeks, even as far back as the time of Homer, had reduced physical training to a system in which agility was cultivated by gymnastic feats and endurance was fostered by athletic contests. The object of this system was to render the manhood of a nation as serviceable as possible for the purposes of warfare. The Romans, as well as all successive dominant peoples down to the end of the middle ages, pursued the same system and with the same intent. Every man in every State was expected to be able to fight, and whilst his hand and his eye were tutored to the skilful use of weapons, so was his general body inured to perfect hardihood by regulated physical training. With the general introduction of firearms and the consequent disuetude of hand-to-hand combat, personal strength began to lose its importance; hence during the next couple of hundred years physical training gradually waned from service. At the beginning of the nineteenth century, however, when the increasing range and precision of firearms entailed greater mobility of infantry, physical training was again introduced into the Prussian Army, an example that was in due course followed by Sweden, by France, and ultimately by England. From the earliest ages up to the middle of the nineteenth century it can be seen that physical training had been merely a military matter, but the altered circumstances of modern life caused it to be also adopted as a civilian necessity. Compulsory bodily exertion was gradually diminishing. The invention of mechanical means of locomotion had led to even trivial journeys being undertaken in the inactive comfort of a railway carriage rather than on horse or on foot. And the tendency of the population to herd within the slums and precincts of great towns deprived the upcoming generation of that active liberty of life which their forbears had been compelled to enjoy. It soon became clear, therefore, that unless this obligatory lethargy could in some way be withstood, physical degeneration was likely to ensue. The remedy was sought in regulated physical exercises.

Athletics and gymnastics began to be cultivated as part of the educational curriculum in schools for sons of the wealthy, whilst calisthenics, with their more suitable mildness of movement, were introduced into seminaries for young ladies. Nor did these advantages long remain the sole privileges of the children of the rich. The Education Act of 1870 and its successors prescribed that some form of physical culture should be imperative on each child educated at the primary schools. Now, as the children at these schools number at any time a seventh of the entire population of the British Isles, and as they have done so for more than a quarter of a century, it can readily be estimated that at least two-thirds of the whole community have enjoyed the presumed benefits of physical culture.

Having set forth these data, one may now turn to an entirely different subject. It has been asserted of late, by the protagonists of what may be termed the Gymnastic School, that physical exercises will not only inhibit the accidence of spinal curvatures, but also that, when such curvatures may have arisen, they can be rapidly and practically cured by a rightly directed course of exercises. Before considering this as a fallacy it may be well to briefly review the established treatment of spinal curvature itself. It was no newly investigated malady; on the contrary, it had been written about centuries before some present-day diseases were even known.

Hippocrates had described it four hundred years before the Christian era, and had laid down its directional diagnosis, as well as its appropriate mechanical treatment. It is true that with the progress of science curvatures began to be discriminated rather by their causes of origin than their direction of curve. Ambroise Paré forecast this distinction in the sixteenth century, and Percivall Pott, by his writings in the eighteenth century, separated carious deformity once and for all from curvatures of the more ordinary types. And it is true, also, as was indeed natural, that the mechanical means employed for treatment underwent improvements that were commensurate with those of other branches of mechanical science. The crude apparatus of Hippocrates, which was not attached to the body, became replaced by appliances that were continuously worn as garments on the body itself. Such appliances were introduced and described by Paré, by Glisson, and by Heister in their respective centuries, and culminated in the invention of Le Vacher, from which the modern appliances have step by step been evolved to their present-day perfection. But, notwithstanding all these advancements both in diagnosis and in treatment, the same general mechanical principle was maintained throughout; a principle that has been, perhaps, most aptly expressed in the dictum of Andry, the father of modern orthopædy, who asserted that "the same method must be used in recovering the shape (of the body) as is used for making streight the crooked trunk of a young tree." Or, in other words, the reduction of the deformity depends on drawing the perverted body towards something that is straighter and stronger than itself.

Now, until about the year 1870, this principle had always been effectually carried out by mechanical means. Its obvious success depended as much on the skill of the mechanician as it did on the ability of the surgeon, and it required so much experience that its practice had become a special one. In the early 'seventies a feeling arose, and it was particularly fostered by certain clinical surgeons at the general hospitals, that if any method of treatment could be discovered that would eliminate the mechanician altogether, and that could also be personally applicable by any and every surgeon to his own cases, then a distinct advantage would be attained. As demand is the constant precursor to supply, it was not long before such a novel method was propounded. It had occurred to the fertile imagination of Dr. Sayre, of New York, that the splint of plaster of Paris bandages which had for some time been successfully used in cases of spinal caries, could, by a modified plan of application, be of equal service in cases of lateral curvature, and he devised his procedure accordingly. The introduction of this method is sufficiently recent for its details to be familiar. The body was brought by suspension into a presumed position of amelioration, and was then encased in bandages of solid plaster. Never perhaps did any new treatment receive so immediate and so universal a trial, and never perhaps did any new treatment come to so sudden a collapse. After several years of complete usage its final results fell under the consideration of the International Medical Congress of 1882, and it was unanimously condemned as useless. Nor were the reasons of failure far to seek. It had been asserted that the jacket, by its pressure over the prominences of the deformity, maintained an improved position of the body, an assertion that was easily controverted. And even had the jacket brought pressure to bear on the prominences of the deformity, it was quite obvious that it also brought equal pressure to bear over the recessions of the deformity, or, in other words, there was no room within the jacket into which the body could straighten.

After the collapse of Sayre there was a pause of several years in all controversy, and for the time being the mechanical method resumed its former position. This did not, however, last for long, seeing that the very same surgeons who had been actively responsible for the introduction of the plaster jacket again sought some other treatment that could be advanced as alternative to the mechanical one. The gymnastic method, which had been previously tried in the 'fifties, and had then faded away into disuse, was in 1886 resuscitated, and its assumptions were embodied in propositions that were tolerably sweeping. It was asserted that by exercises a lateral curvature without osseous deformity could be cured within a month. And it was also asserted that although a curvature with osseous deformity was incurable, yet it could be brought within three months into a best possible position which would then remain permanent.

These assertions the adherents of the Mechanical School entirely traversed. They affirmed that where there was no osseous deformity there was no actual curvature at all, and that therefore such ordinary slouches of figure as were familiar in a raw recruit were, of course, removable by simple drill. And they affirmed, also, that where there was actual and osseous curvature, a thing then admitted by the Gymnastic School to be incurable, there was the ample possibility of complete cure in moderate cases by the mechanical method, that is to say, by the upbuoying of the trunk and by the exercise of active forces over the prominences of the deformity. And they, moreover, affirmed that even the very worst and most neglected cases could, at all events with time, be brought into a good general restoration of bodily form, even although some curvature might still remain existent in the spine itself. Finally, they affirmed that as the period of cure must of necessity be co-relative to the period of lapse, the three months' limitation of the Gymnastic School was an entirely unreasonable one.

In this position things remained at issue for the next ten years. During that time the importance of general physical culture was being rightly and strenuously advocated for the up-growing generation, and nothing, therefore, was simpler than that the gymnastic treatment of curvature should be confused with it, although the two matters were alien one to another. Hence the Gymnastic School, far from holding to its previous assertions, formulated a fresh proposition that was still more sweeping. It promised that all ordinary cases of lateral curvature could be practically cured within three months. And it promised, moreover, to produce tabulated statistics in confirmation of this somewhat astounding statement. These statistics have in due course come to hand, and, after careful analysis, have been found to disprove the very facts they were advanced to support. In the first place, they fail to record or even to claim any cases of cure at all. All they actually do is to assert improvement, and to endeavour to substantiate this by an appeal to those metal-strip tracings that had already been condemned as valueless by the International Medical Congress. In the next place, it is evident, and also from the same statistics, that even over a long period of years very few of those medical men who had ventured to give the gymnastic treatment a single trial ever pursued it further. The statistics themselves, therefore, prove to be entirely contradictory on the very points for which they have been adduced.

Now with a deformity like spinal curvature, the proofs of the value of any curative method must practically rest on two distinct sets of evidence. The one is the extent to which such a method has been adopted by recognised authorities after its purely experimental stages have been passed. And the other is the value that can be laid on the personal records of those that practise it.

With the gymnastic treatment it can be seen that it fails in these respects. With the mechanical treatment, on the other hand, the reverse has evidently been the case. It is certainly obvious that the latter method has long since passed its experimental stage. And it can certainly be proved that the greatest consulting surgeons of their time (as Sir Astley Cooper in one generation, and Sir James Paget in the next, to say nothing of others) dealt with their spinal curvature cases over many years, and to numbers that ran into thousands, by the mechanical method —a thing they would scarcely have done unless they had been convinced by long experience of its efficacy.

As to present-day records of proof, the Mechanical School relies upon photographs taken in series during the course of each case, and always under constantly fixed conditions of lens, light, focus, and position. These, at all events, if the negatives are left untouched, must be reliable; whereas the metal-strip tracings which are used by the Gymnastic School have long since been proved to be fallacious.

The truth is that in the popular mind, the gymnastic treatment of curvature has lately been vaguely confused with ordinary physical culture. The latter, as has been shown, was for centuries employed as a military factor in the perfection of soldiery. It had, under the new conditions of modern life, been advantageously brought into civilian use for the bodily development of up-growing boys and girls. The beneficial results derived from it in these respects were indubitable, despite the present-day allegations of physical degeneration.

But physical culture, like all things else, has its limitations. When it was suggested, a quarter of a century since, that if physical culture could be made universal, lateral curvature would cease to arise, there were only theoretical grounds on which to rebut this proposition. But to-day the working of the Education Acts have produced a practical refutation. Two-thirds of the entire population have already enjoyed the benefits of physical culture, and to-day spinal curvature shows not the least signs of diminution. It is therefore perfectly clear that gymnastic exercises will neither inhibit the incidence of spinal curvatures, nor are they capable of curing actual curvatures when these have once arisen.

